# Genetic diversity and structure of *Bipolaris oryzae* and *Exserohilum rostratum* populations causing brown spot of rice in Burkina Faso based on genotyping-by-sequencing

**DOI:** 10.3389/fpls.2022.1022348

**Published:** 2022-11-25

**Authors:** Kouka Hilaire Kaboré, Abalo Itolou Kassankogno, Henri Adreit, Joëlle Milazzo, Sonia Guillou, Laurence Blondin, Laurie Chopin, Sébastien Ravel, Florian Charriat, Mariam Barro, Charlottte Tollenaere, Marc-Henri Lebrun, Didier Tharreau

**Affiliations:** ^1^ Université Paris-Saclay, INRAE, UR BIOGER, Palaiseau, France; ^2^ CIRAD, UMR PHIM, Montpellier, France; ^3^ CNRST/INERA, Laboratoire de Phytopathologie, Bobo-Dioulasso, Burkina Faso; ^4^ PHIM, Univ Montpellier, CIRAD, INRAE, IRD, Montpellier SupAgro, Montpellier, France

**Keywords:** linkage disequilibrium, mating type, recombination, reproduction, rice disease, fungal pathogen

## Abstract

In recent years, Brown spot disease of rice (BSR) has been observed on leaves and seeds of rice in all rice-growing areas of Burkina Faso. *Bipolaris oryzae* and *Exserohilum rostratum* are the main fungal species isolated from BSR infected tissues and they are frequently observed in the same field. However, we are lacking information on the genetic diversity and population structure of these fungi in Burkina Faso. The mode of reproduction is also unknown. The genetic diversity of isolates of *B. oryzae* (n=61) and *E. rostratum* (n=151), collected from major rice-growing areas of Burkina Faso, was estimated using genotyping-by-sequencing (GBS). The mean values for nucleotide diversity (π) were 1.9 x10^-4^ for *B. oryzae* and 4.8 x10^-4^ for *E. rostratum*. There is no genetic differentiation between the geographical populations of each species. The analysis of molecular variance revealed that 89% and 94% of the genetic variances were within the populations of *B. oryzae* and *E. rostratum*, respectively. For each species, four genetic clusters were identified by two clustering methods (DAPC and sNMF). The distribution of these genetic groups was independent of the geographical origin of the isolates. Evidence of recombination was detected in the populations of *B. oryzae* and *E. rostratum.* For *B. oryzae* balanced mating type ratios were supporting sexual reproduction. For *E. rostratum* overrepresentation of MAT1-2 isolates (79%) suggested a predominant asexual reproduction. This study provides important information on the biology and genetics of the two major fungi causing brown spot disease of rice in Burkina Faso.

## Introduction

Rice is the second most important source of food after corn in Sub-Saharan Africa (SSA, Nigatu et al., 2017). However, SSA rice cropping is characterized by a low average yield of 1.4 t/ha, compared to over 4 t/ha in Asia (Nwanze et al., 2006). Local rice production cannot meet the needs, leading to import up to half 50% of the rice consumed. The major constraints significantly reducing rice production in SSA are drought, iron toxicity, salinity, weeds, rodents, birds and diseases (Diagne et al., 2013; van Oort, 2018).

Among diseases, Brown spot of rice (BSR) is becoming a threat in SSA. This disease is expanding, and is currently estimated to be responsible for the loss of 3.3% of the regional rice production (Savary et al., 2019). BSR occurs as brown spots on coleoptiles, leaf blades, sheaths, stems, glumes and seeds (Ou, 1985; Sunder et al., 2014). The disease is favored by mineral deficiencies in the soil, especially nitrogen, high temperatures and lack of water (Zadoks, 1974; Ou, 1985; Savary et al., 2000; Savary et al., 2005).

In Burkina Faso, the importance of BSR in rice nurseries and fields was already highlighted in 1962 (Davet, 1962). An outbreak of BSR was recorded in 1994 in the most important rice-growing plain of the country (Sourou) and, since then, the disease has spread to other rice-growing areas (Ouédraogo, 2008). Under natural disease pressure, grain yield losses could reach 16% (Ouédraogo, 2008). A recent study has shown that 80% of rice fields in western Burkina Faso displayed BSR leaf symptoms (Barro et al., 2021). Furthermore, up to 76% of rice grains produced in Burkina Faso are infected (Ouédraogo et al., 2016). This high prevalence leads to reconsider the status of BSR and justifies to improve knowledge of the pathogen biology to ameliorate control methods.

Several species of fungi such as *Bipolaris oryzae, B. bicolor, B. indica, B. victoriae*, *B. zeicola* (Motlagh and Kaviani, 2008; Aslam et al., 2021) and *Exserohilum rostratum* (Majeed et al., 2016; Toher et al., 2016; Kaboré et al., 2022) are responsible for BSR. In Burkina Faso, *Bipolaris oryzae* (syn. *Cochliobolus miyabeanus*) and *Exserohilum rostratum* (syn. *Setosphaera rostrata*), are the two species most frequently isolated from BSR infected tissues (Kaboré, 2022). These species are frequently found in the same rice fields or in the same seed lots (Ouédraogo et al., 2016; Kaboré, 2022).

*Bipolaris oryzae* and *E. rostratum* are heterothallic fungi, that is, their sexual reproduction requires strains of opposite mating types (MAT1-1 and MAT1-2) (Ueyama and Tsuda, 1976). Most studies on *B. oryzae* populations have reported a high level of genetic and genotypic diversity and low geographical population structure (Castell-Miller and Samac, 2012; Archana et al., 2014a; Ahmadpour et al., 2018). Sexual reproduction of *B. oryzae* was suspected based on equal frequencies of mating types, but recombination was not confirmed by estimating linkage disequilibrium (Castell-Miller and Samac, 2012; Ahmadpour et al., 2018). *Bipolaris oryzae* isolates from Burkina Faso showed significant morphological and pathological variability (Ouédraogo et al., 2004; Dembélé, 2014; Boka et al., 2018). Investigations based on mitochondrial DNA RFLP markers revealed the division of *B. oryzae* isolates into two haplotypes (Ouédraogo et al., 2004).

In the field, the infection cycle of *B. oryzae* starts with the deposition of asexual spores (conidia), on aerial parts of rice. Conidia produce a germ tube that adhere to the plant surface thanks to a mucilaginous substance. Then, an appressorium is formed that allows direct penetration of cells (Hau and Rush, 1982; Ou, 1985). After tissue colonization by mycelial hyphae, lesions appear 18 to 36 hours post-infection (Tullis, 1935; Johnson and Percich, 1992; Dallagnol et al., 2009). In cultivated rice, a peak of conidia production from new lesions is observed 6 days after infection (Ou, 1985; Barnwal et al., 2013). Less is known about the infection cycle of *E. rostratum* on rice, but a recent histopathological study revealed that on contact with rice leaves, *E. rostratum* conidia produce appressoria within 24 h that directly penetrate the epidermal cells with the onset of symptoms within 3 days (Korra et al., 2022).

To date, the genomics studies of *B. oryzae* and *E. rostratum* are also limited. The genome sequence of a strain of *E. rostratum* isolated from maize (BioSample: SAMN19599129) was released recently. The description of the complete circular mitochondrial genome of *E. rostratum* (ZM170581) isolated from maize followed (Ma et al., 2022). Genomes of *E. rostratum* strains (BioSamples: SAMN14931704 and SAMN30522240) isolated from rice have also been sequenced recently (https://www.ncbi.nlm.nih.gov/biosample/). For *B. oryzae* a reference genomes are available from a strain isolated from cultivated rice (Condon et al., 2013) and a strain isolated from wild rice (*Zizania palustris*) (Castell-Miller et al., 2016). These data were used for genomic structure studies (Condon et al., 2013; Ma et al., 2022).

However, there is a lack of information on the genetic structure and diversity, as well as the mode of reproduction of *B. oryzae* in rice fields in Burkina Faso. Our knowledge of *E. rostratum* in Burkina Faso is limited since no population study was performed to date. The objectives of this study were to: (i) assess the genetic diversity, (ii) describe the genetic structure, and (iii) determine the mode of reproduction of *B. oryzae* and *E. rostratum* populations from major rice-growing areas of Burkina Faso.

## Materials and methods

### Sample collection and isolation of strains

**Figure 1:**
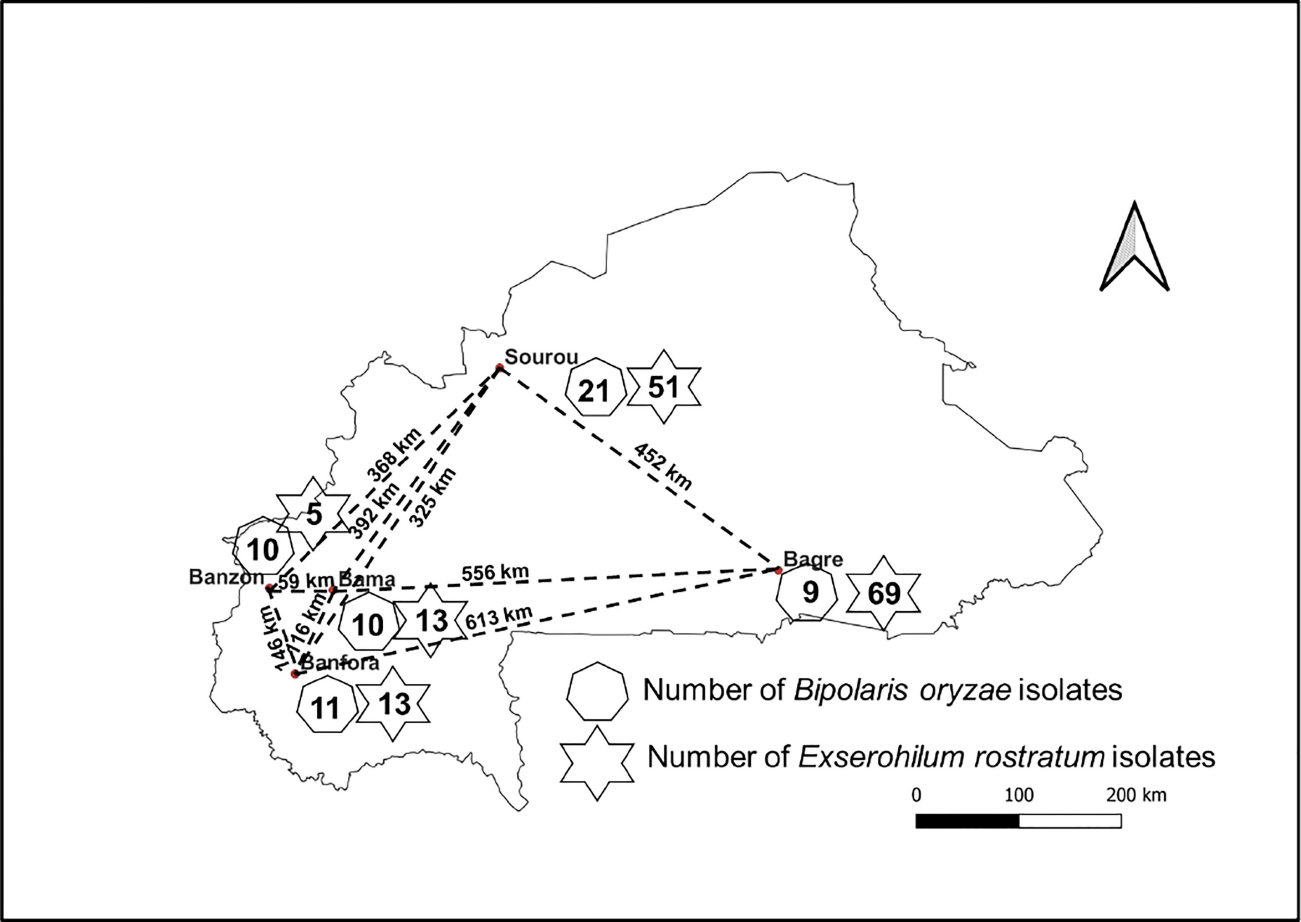
Schematic location of collection sites for infected rice samples. The number of *Bipolaris oryzae* and *Exserohilum rostratum* isolates isolated is indicated by the heptagons and stars respectively. Names in bold refer to the localities where samples were collected and the dashed lines indicate the distance between collection sites.

Leaves and seeds of cultivated rice (*Oryza sativa*) showing symptoms of BSR were collected in 2018 and 2019 in different rice fields from Bagré, Bama, Banfora, Banzon and Sourou (**Figure 1**; **Supplementary Tables S1, S2**). These sites are on average 365 km apart (minimum distance 59 km and maximum distance 622 km) and represent the main rice-growing areas of Burkina Faso. Diseased leaves and seeds were arranged in Petri dishes containing a double layer of moistened blotting paper (Mathur and Kongsdal, 2003). Incubation was carried out at 25°C for 7 days under alternating 12 h of white light and 12 h of darkness. Using a flame-stretched Pasteur pipette, fungal conidia formed in the lesions were collected and spread on bacto-agar medium. After incubation for 24 h (25°C, 12 h white light and 12 h darkness), a single germinating conidium was transferred with a sterile scalpel onto potato dextrose agar medium (PDA, 39 g/L) and incubated under the same conditions for 7 days.

### DNA extraction

Genomic DNA was extracted from fresh mycelium with modifications to the cetyl trimethyl ammonium bromide (CTAB) method described by Doyle and Doyle (1987). A 5-day-old fragment of mycelium was transferred onto Corn Meat Agar (CMA, 18 g/L) medium previously covered with a sterile cellophane disc. The plates were incubated at 25°C (12 h photoperiod) for 7 days, the mycelium was scraped off with a scalpel and approximately 30 mg of the mycelium was transferred to a 2 mL tube containing sterile sand and 2 iron beads. These tubes were placed directly into liquid nitrogen and the mycelium was cold ground using a Retsch mill (Retsch MM 301) for 4 min at 15 Hz. After grinding, each tube received 1 mL of CTAB lysis buffer with 1% polyvinyl pyrrolidone (MW. 360 000), 1% Na_2_SO_3_, 0.2 mg/mL RNase and 1 mg/mL proteinase K. The tubes were vortexed (Vortex-Genie 2) and incubated in the oven at 65°C for 2 h. The tubes were then centrifuged for 3 min at 8,000 rpm and approximately 900 µL of the supernatant was transferred to a new 2 mL tube. One mL of chloroform isoamilalcohol (CIAA, 24/1 v/v) was placed in each tube and mixed by inversion before being centrifuged at 10,000 rpm for 10 min. After centrifugation, 700 µL of the supernatant was transferred to 1.5 mL tubes. Six hundred µL of isopropanol cooled to -20°C and 210 µL of 5M NaCl were added and mixed by inversion. The tubes were kept at -20°C overnight. They were then centrifuged at 4°C for 30 min at 13,000 rpm and the supernatant was removed by inverting the tubes. Five hundred µL of 70° ethanol was added to each tube and centrifuged at 4°C for 5 min at 13,000 rpm. Ethanol was removed by inverting the tubes and draining on absorbent paper. The tubes were dried for 1 h in the fume cupboard. DNA was re-suspended in 100 µL of sterile milliQ water. The quality and quantity of the extracted DNA was assessed by electrophoresis in 1% (w/v) agarose gels and spectrophotometry (Nanodrop™ 2000).

### Preparation of genotyping-by-sequencing libraries

The preparation of GBS libraries was carried out in collaboration with the genotyping platform of the UMR AGAP/CIRAD according to the method described by Elshire et al. (2011). Genomic DNA of the strains were normalized to a concentration of 10 ng/µL and digested with the restriction enzyme *Ape*KI (5 U) at 75°C for 2 h. The restriction digestion of DNA was followed by ligation with adapters. The adapters included different barcodes for tagging individual samples and common adapters. The ligation was performed using T4 DNA ligase at 22°C for 30 min and the ligase was inactivated by holding at 65°C for 10 min. Two pools were created. To assess the reproducibility of GBS, each pool contained 6 randomly selected and duplicated isolates. Ligation products were purified with the QIAquick purification kit from QIAgen. The library was enriched by PCR to 50 µL of reaction solution including 25 µL of 2X Taq Master mix NEB, 19 µL of water, 2 µL of DNA and 2 μL each of the forward and reverse primers (10 μM). Amplification was done according to the following program: 98 ˚C for 30 s, followed by 18 cycles of 98 ˚C for 10 s, 65 ˚C for 30 s, 72 ˚C for 30 s, ending at 72 ˚C for 5 min. PCR products were purified with QIAgen’s QIAquick Purification Kit before being evaluated with BioAnalyzer 2100 (Agilent Technologies) for fragment size distribution. The GBS libraries were sequenced on an Illumina HiSeq 4000 producing 150 bp paired-end reads.

### Sequence preprocessing and SNP identification

The bioinformatics tool RattleSNP (https://github.com/sravel/RattleSNP) was used to process the data. The workflow performs demultiplexing, sequence mapping to the reference genome and variant calling. The sequences of *B. oryzae* isolates were mapped onto the genome of strain ATCC 44560 (Condon et al., 2013), and those of *E. rostratum* onto the genome of BF9006 sequenced by CIRAD (https://www.ncbi.nlm.nih.gov/biosample/SAMN30522240/). Prior to variant calling, strains with a mapping rate of less than 75% to the reference genome, a library size of less than 100,000 reads and a sequencing depth of less than 3 x were considered low quality data and excluded from the dataset. SNPs from strains meeting the criteria (61 isolates for *B. oryzae* and 151 isolates of *E. rostratum*) were filtered for missing rate (–max-missing 0.70), minor allele frequency (–maf 0.05), depth (–minDP 2) and site quality (–minQ 30) using VCFtools version 01.16 (Danecek et al., 2011)

### Population structure and genetic differentiation analyses

Discriminant Analysis of Principal Components (DAPC) (Jombart et al., 2010) and sNMF (Frichot et al., 2014) analysis were used to investigate the genetic structure of these fungal populations. DAPC was carried out with the adegenet package of the R software. First, the find.clusters function was used to detect the number of clusters in the population (Jombart, 2008; Jombart et al., 2010). The function uses K-means clustering which decomposes the total variance of a variable into two components: inter-group and intra-group. This model maximizes the inter-group variation (Jombart et al., 2010).

sNMF of the LEA R software package was used to estimate the number of discrete genetic clusters (K) and admixture coefficients (Frichot et al., 2014). The optimal number of sNMF clusters was defined as the one corresponding to the lowest cross-entropy value.

To further characterize the genetic structure of the population described by the DAPC and sNMF analyses, a hierarchical analysis of molecular variance (AMOVA) (Excoffier et al., 1992) were performed with the poppr package of R (Kamvar et al., 2014). Pairwise genetic differentiation (F_ST_) was calculated using the ‘genet.dist’ function in the hierfstat package of R (Goudet, 2005), following the method described by Weir and Cockerham (1984). RAxML (Randomized Axelerated Maximum Likelihood) version 8.2.12 was used to assess phylogenetic relationships between isolates using the maximum likelihood method (Stamatakis, 2014).

### Genetic and genotypic diversity analyses

Analysis of genotypic diversity and association indices of fully or partially clonal populations is recommended before and after clone correction (Milgroom, 1996; Grünwald and Hoheisel, 2006; Kamvar et al., 2014). To identify clones, the dissimilarity rate between duplicates of the same isolate was calculated using the *bitwise.dis* function of the R poppr package (Kamvar et al., 2014). For a given species, the maximum dissimilarity between duplicates was measured and used as a threshold to define clones. Two isolates with a dissimilarity rate lower than the threshold were considered as clones.

To estimate genotypic diversity, the poppr package of R (Kamvar et al., 2014), was used to calculate Shannon-Wiener index (H) (Shannon, 1948), Simpson’s complement index of genotypic diversity index (λ) corrected for sample size (Simpson, 1949; Grünwald et al., 2003) and evenness (E5) (Pielou, 1975; Ludwig and Reynolds, 1988; Grünwald et al., 2003). To adjust for the population size, the raw value of the Simpson’s complement index of genotypic diversity index was multiplied by N/(N-1) with N being the number of isolates in the population (Grünwald et al., 2003).

To assess gene diversity, nucleotide diversity (π) values were calculated with VCFtools across the genome with 10 kb windows (Danecek et al., 2011).

### Linkage disequilibrium and recombination

Detection of recombination was carried out by calculating linkage disequilibrium (LD) and clonality was tested with the PHI test from genotyping data. Pairwise LD between loci was calculated based on allelic frequency correlations (r^2^) over a 10 kb window using the PopLDdecay program (Zhang et al., 2019).

A FASTA file was used to generate a NeighborNet in SPLITSTREE4 and the PHI test was performed to test the null hypothesis of clonality (Huson and Bryant, 2006).

### Mating type characterization and *in vitro* sexual reproduction

Identification of mating types was done by amplification of MAT1-1 and MAT1-2 genes with primers specific to *B. oryzae* (Ahmadpour et al., 2018) and *E. rostratum* (Kusai et al., 2016). Each PCR reaction using the kit Go-Taq (Promega) had a final volume of 25 μL, containing 1 μL of template genomic DNA (20 ng/µL),1 μL forward primer (10 µM), 1 μL reverse primer (10 µM), 1µL of dNTPs (10 µM each), 5 μL of the buffer 5x, 0.25 µL Taq polymerase (5U/µL) and 14.75 μL sterile water. For the amplifications of *E. rostratum* MAT genes, the amount of water was 13.75 µL and 1 µL MgCl_2_ 5X was added. PCR cycling was performed as follows: 94°C for 2 min; followed by 35 cycles of 94°C for 1 min, 52°C for 1 min and 72°C for 1 min; plus a final extension at 72°C for 7 min for *B. oryzae.* For *E. rostratum*, initial denaturation at 95°C for 2 min; followed by 35 cycles of denaturation at 95 C for 30 s, annealing at 60°C for 30 s, extension at 72°C for 1 min; and final extension at 72°C for 5 min. The null hypothesis of a 1:1 ratio between mating types was tested using the chi-square goodness-of-fit.

The sexual fertility of *B. oryzae* and *E. rostratum* isolates was tested *in vitro* according to the protocol described by Tsuda and Ueyama (1976). A dry, autoclaved rice leaf of about 4 cm was placed in the center of a petri dish on Sach’s culture medium. Pieces of mycelium from the pre-determined MAT1-1 and MAT1-2 strains were placed on either side of the rice leaf. The dishes were sealed with tape to prevent dehydration of the medium and then placed in an oven at 24°C for 30 days.

## Results

### Genetic structure of *B. oryzae* and *E. rostratum* populations

The genetic diversity of isolates of *B. oryzae* (n=61) and *E. rostratum* (n=151) collected in Burkina Faso, was estimated using genetic markers (SNPs) obtained by genotyping-by-sequencing. After alignment to the *B. oryzae* ATCC 44560 reference genome composed of 619 scaffolds and filtering, 28,907 SNPs were identified in the *B. oryzae* samples (61 isolates). Sequences of *E. rostratum* isolates (n=151) aligned to the BF9006 isolate reference genome (377 scaffolds) yielded 102,464 SNPs.

To study the *B. oryzae* and *E. rostratum* populations from Burkina Faso, the isolates were clustered by DAPC according to their multilocus genotypes. Inference of ancestry was also performed with sNMF.

**Figure 2:**
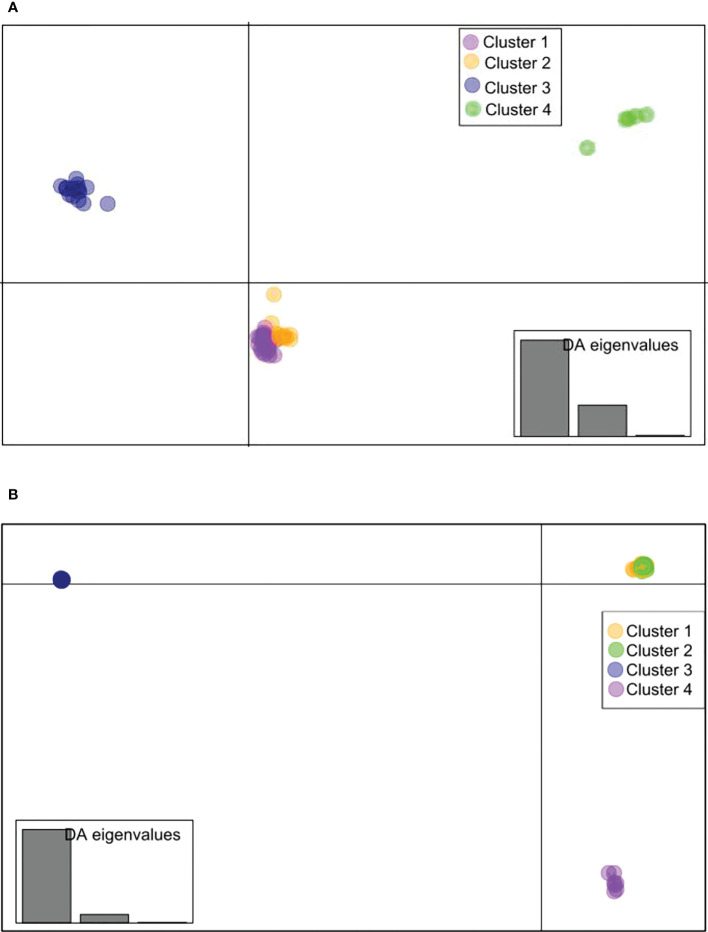
Discriminant analysis of principal components (DAPC) scatterplot drawn using SNPs in the R package adegenet (K =4). **(A)** *Bipolaris oryzae*, **(B)** *Exserohilum rostratum*. Each color represents a cluster obtained by DAPC analysis of isolates from different localities in Burkina Faso. For *Bipolaris oryzae*, Cluster 1 is composed of 27 isolates (Bagre = 4, Bama = 3, Banfora = 5, Banzon = 8 and Sourou =7), Cluster 2 is composed of 13 isolates (Bagre = 4, Bama = 3, Banfora = 1, Banzon = 2 and Sourou =3), Cluster 3 is composed of 16 isolates (Bagre = 1, Bama = 4 and Sourou =11), Cluster 4 is composed of 5 isolates (Banfora = 5). For *Exserohilum rostratum*, Cluster 1 is composed of 58 isolates (Bagre = 38, Bama = 4, Banfora = 4, Banzon = 2 and Sourou =10), Cluster 2 is composed of 60 isolates (Bagre = 23, Bama = 4, Banfora = 7, Banzon = 2 and Sourou =24), Cluster 3 is composed of 26 isolates (Bagre = 5, Bama = 5, Banfora = 2 and Sourou =14) and Cluster 4 is composed of 7 isolates (Bagre = 3, Banzon = 1 and Sourou = 3).

**Figure 3:**
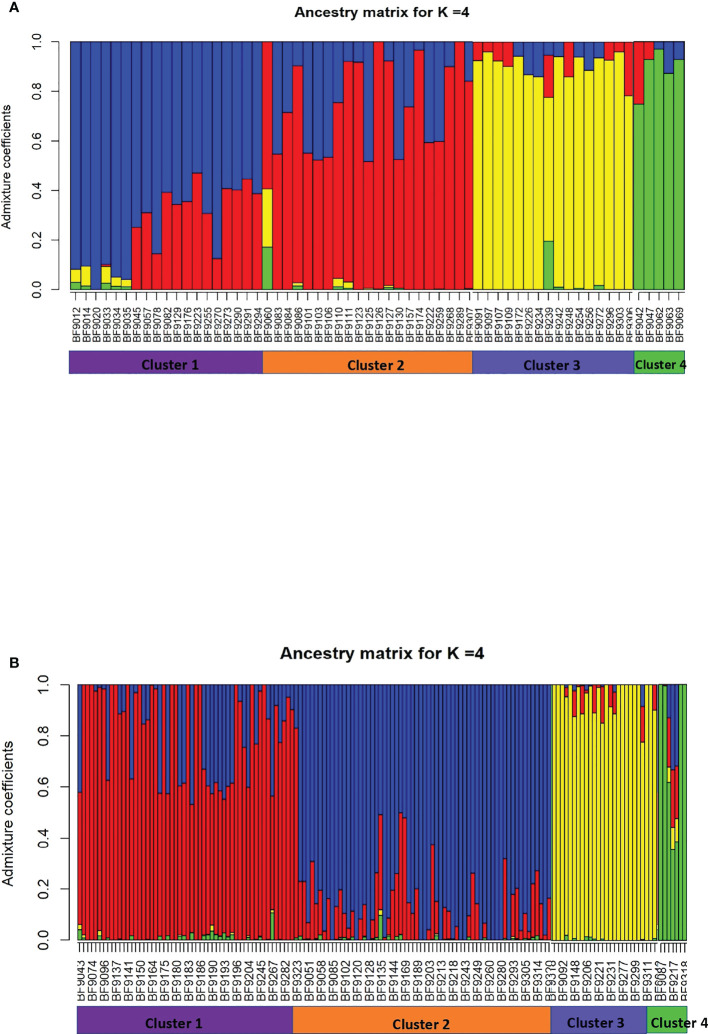
Population structure analysis of *Bipolaris oryzae* and *Exserohilum rostratum*. Ancestry coefficient for each isolate calculated by using sNMF with K=4. **(A)** *B. oryzae*, **(B)** *E. rostratum.*.

**Figure 4:**
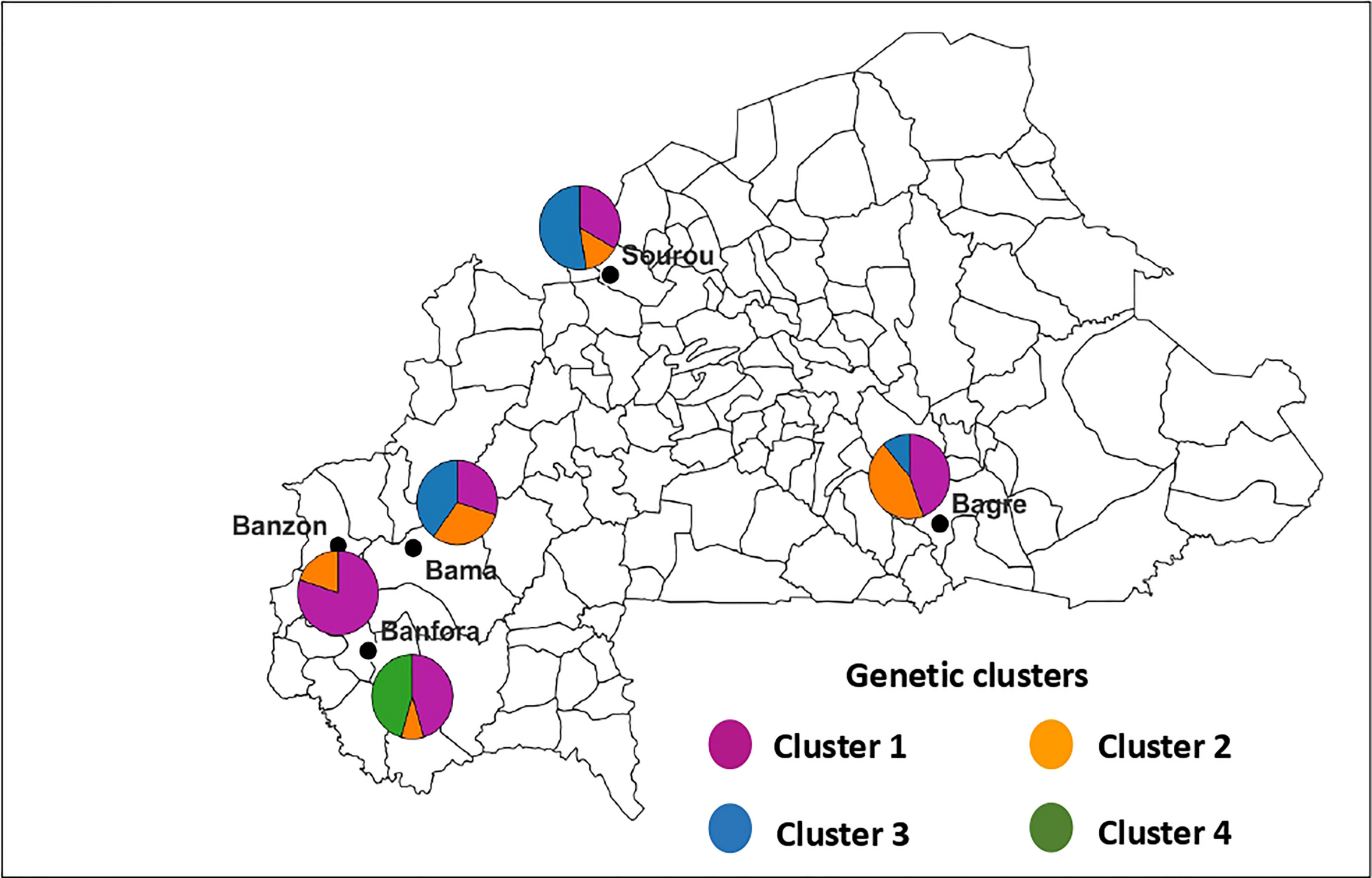
Geographic distribution of the four genetic clusters of *Bipolaris oryzae* in Burkina Faso. The size of sectors is proportional to the proportion each cluster defined by DAPC in each area. Cluster 1 (n = 27 isolates), Cluster 2 (n = 13 isolates), Cluster 3 (n = 16 isolates) and Cluster 4 (n = 5 isolates).

For *B. oryzae*, the bayesian information criterion (BIC) of DAPC suggested the best clustering for four genetically distinct populations (K = 4; **Supplementary Figure S1**, **Figure 2A**). Similarly, the sNMF analysis indicated an optimum for four clusters (**Supplementary Figure 2**, **Figure 3A**). The phylogenetic tree made with RaxML also highlighted four clades (**Supplementary Figure S3**). The clustering of isolates according to DAPC, sNMF and RaxML was similar. Isolates from cluster 1 and 2 were found in all five geographical collection areas (**Table 1**, **Figure 4**), while cluster 4 was only found in Banfora. Isolates from cluster 3 were detected in three areas namely Bagré, Bama and Sourou. The sNMF analysis showed a significant admixture between the four clusters, but particularly between cluster 1 and 2 (**Figure 3A**). The F_ST_ values between the *B. oryzae* isolates from the different rice-growing areas in Burkina Faso showed little differentiation between sites, ranging from 0.005 to 0.132 (**Table 2**) and with an average value of 0.08. No correlation was observed between geographic distances of rice growing sites and genetic differentiation index values (**Supplementary Figure S4**). The AMOVA analysis revealed that the variability was 88.65% within localities and 11.35% among the five rice-growing zones (**Table 3**).

**Table 1:** Distribution of *Bipolaris oryzae* and *Exserohilum rostratum* clusters obtained by DAPC analysis in five rice-growing areas of Burkina Faso.

Localities	*Bipolaris oryzae* clusters				*Exserohilum rostratum* clusters			
	1	2	3	4	1	2	3	4
Bagré	4	4	1	0	38	23	5	3
Bama	3	3	4	0	4	4	5	0
Banfora	5	1	0	5	4	7	2	0
Banzon	8	2	0	0	2	2	0	1
Sourou	7	3	11	0	10	24	14	3
Total	27	13	16	5	58	60	26	7

**Table 2:** Pairwise F_ST_ estimates between *Bipolaris oryzae* isolates from five rice-growing zones in Burkina Faso.

	Bagré	Bama	Banfora	Banzon
Bama	0.030			
Banfora	0.116	0.086		
Banzon	0.066	0.113	0.125	
Sourou	0.064	0.005	0.079	0.132

**Table 3:** Analysis of molecular variance (AMOVA) of *Bipolaris oryzae* among and within five rice-growing zones in Burkina Faso.

		df	Sum Sq	Mean Sq	Sigma	%	P value
Localities							
	Variation among localities	4	26214.2	6553.55	334.25	11.35	0.0004
	Variation within localities	56	146219	2611.05	2611.05	88.65	
	Total	60	172433.2	2873.89	2945.3	100	
Genetic clusters							
	Variation among clusters	3	72923.27	24307.76	1624.25	48.2	0.0001
	Variation within clusters	57	99509.97	1745.79	1745.79	51.8	
	Total	60	172433.24	2873.89	3370.4	100	

Sq, Sum of squares; %, Percentage of variation.

**Figure 5:**
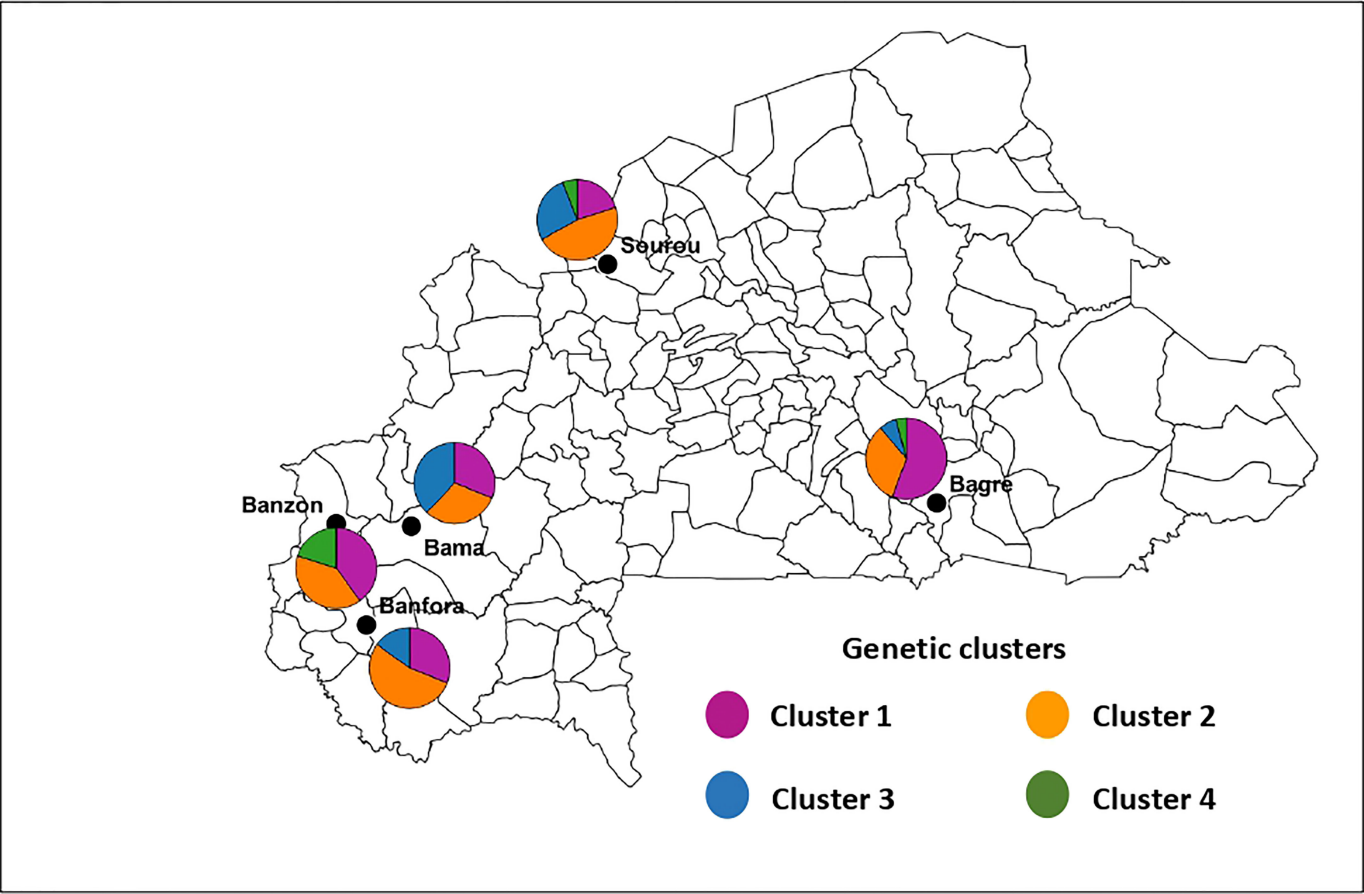
Geographic distribution of the four genetic clusters of *Exserohilum rostratum* in Burkina Faso. The size of sectors is proportional to the proportion each cluster defined by DAPC in each area. Cluster 1 (n = 58 isolates), Cluster 2 (n = 60 isolates), Cluster 3 (n = 26 isolates) and Cluster 4 (n = 7 isolates).

For *E. rostratum*, the bayesian information criterion (BIC) of DAPC suggested the best clustering for four genetically distinct populations (K = 4; **Supplementary Figure 5**, **Figure 2B**). Similarly, the sNMF analysis indicated an optimum of four clusters (**Supplementary Figure S6**; **Figure 3B**). The phylogenetic tree made with RaxML also highlighted four clades (**Supplementary Figure S7**). The clustering of isolates was identical between methods. Isolates from clusters 1 and 2 were found in all five localities sampled. Isolates from cluster 3 were found in all localities (**Figure 5**) except Banzon, and isolates cluster 4 was sampled in Bagré, Banzon and Sourou. Very low F_ST_ values ranging from 0 to 0.129 and with an average value of 0.022 were observed between populations of different rice production areas in Burkina Faso (**Table 4**). Distances between the different rice production sites had no impact on population structure (**Supplementary Figure S8**). The AMOVA analysis revealed that the variability was 94.4% within and 5.6% among the five rice-growing areas (**Table 5**).

**Table 4:** Pairwise F_ST_ estimates between *Exserohilum rostratum* isolates from five rice-growing zones in Burkina Faso.

	Bagré	Bama	Banfora	Banzon
Bama	0.129			
Banfora	-0.0013	0.017		
Banzon	-0.0209	0.011	-0.058	
Sourou	0.068	-0.018	-0.001	-0.020

Negative values of F_ST_ are considered as zero values.

**Table 5:** Analysis of molecular variance (AMOVA) of *Exserohilum rostratum* among and within five rice-growing zones in Burkina Faso.

		df	Sum Sq	Mean Sq	Sigma	%	P value
Localities							
	Variation among localities	4	121493.6	30373.40	724.64	5.6	0.01
	Variation within localities	146	1793776.4	12286.14	12286.14	94.4	
	Total	150	1915270	12768.47	13010.78	100	
Genetic clusters							
	Variation among clusters	3	1328106.2	442702.07	13150.78	76.7	0.0001
	Variation within clusters	147	587163.8	3994.31	3994.31	23.3	
	Total	150	1915270	12768.47	17145.09	100	

Sq, Sum of squares; %, Percentage of variation.

### Genotypic and genetic diversity

Samples were analyzed for possible clones. The genetic distance between duplicates of *B. oryzae* isolates varied from 0.035 to 0.087. Those of *E. rostratum* varied between 0.015 and 0.033. *Bipolaris oryzae* and *E. rostratum* isolates were considered as clones if their dissimilarity rates were less than or equal to 0.087 and 0.033 respectively. Based on this criterion, 8 and 11 isolates of *B. oryzae* and *E. rostratum* respectively were considered as clones of two or more isolates. Clones represented 13% (8/61) and 7% (11/151) of the isolates in each sample of *B. oryzae* and *E. rostratum*, respectively (**Table 6**).

**Table 6:** Genotypic and gene diversity indices of *Bipolaris oryzae* and *Exserohilum rostratum* isolates from five rice-growing areas of Burkina Faso.

	*B. oryzae* population						*E. rostratum* population											
	N	Clones	H	Lambda	E.5	π	N		Clones		H		lambda		E.5		π	
Localities																	
Bagre	9	1	2.20	1	1	1.9 x10^-4^	69		6		4.23		0.999		1		5.7 x10^-4^	
Bama	10	1	2.30	0.999	1	2.2 x10^-4^	13		0		2.56		1		1		9.8 x10^-4^	
Banfora	11	2	2.40	0.999	1	1.7 x10^-4^	13		2		2.56		0.999		1		6.8 x10^-4^	
Banzon	10	3	2.30	0.999	1	1.3 x10^-4^	5		1		1.61		1		1		6 x10^-4^	
Sourou	21	1	3.04	0.999	1	2.4 x10^-4^	51		2		3.93		0.999		1		9 x10^-4^	
Genetic clusters																	
Cluster 1	27	4	3.30	1	1	1.5 x10^-4^	58		4		4.0604		0.999		1		3.5 x10^-4^	
Cluster 2	13	2	2.56	0.999	1	1.3 x10^-4^	60		3		4.0943		0.999		1		3.5 x10^-4^	
Cluster 3	16	0	2.77	1	1	1.7 x10^-4^	26		0		3.2581		0.999		1		2.4 x10^-4^	
Cluster 4	5	2	1.61	1	1	1.4 x10^-4^	7		4		1.9459		0.999		1		2.9 x10^-4^	

N: Number of isolates; Clones: number of isolates with a repeated multilocus genotype; H: Shannon-Wiener Index; Lambda: Simpson’s Index; E.5: Evenness (1/λ)−1/e^H^ – 1); π: nucleotide diversity.

Genotypic and gene diversity indexes are presented in **Table 6**. For *B. oryzae*, the genotypic diversity (H) between isolates from different rice-growing areas ranged from 2.20 to 3.04 with an average value of 2.45. These indices are influenced by population size and do not allow comparison between different localities. The Simpson index corrected for sample size ranged from 0.999 to 1 (mean value 0.999) indicating a high level of genotypic diversity within each population. The regularity index (E.5) was equal to 1 for all populations.

In terms of genetic diversity, nucleotide diversity values ranged from 1.3 x10^-4^ to 2.4 x10^-4^ with an average of 1.9 x10^-4^. The Banzon population also showed the lowest value of nucleotide diversity (1.3 x10^-4^). The Sourou population showed the highest diversity (2.4 x10^-4^). It was followed by the populations of Bama, Bagré and Banfora.

For *E. rostratum*, the genotypic diversity (H) between isolates from different rice-growing areas ranged from 1.61 to 4.23 with an average value of 2.98. The Simpson index corrected for sample size ranged from 0.999 to 1 (mean value 0.999) indicating high genotypic diversity within each population. The regularity index (E.5) was equal to 1 for all populations. The values of nucleotide diversity varied between 5.7 x10^-4^ and 9.8 x10^-4^. The average value was 4.8 x10^-4^. The isolates from Bama showed the highest value of nucleotide diversity (9.8 x10^-4^), while the lowest value was observed in Bagré (5.7 x10^-4^).

### Recombination, mating type distribution and *in vitro* sexual reproduction

**Figure 6:**
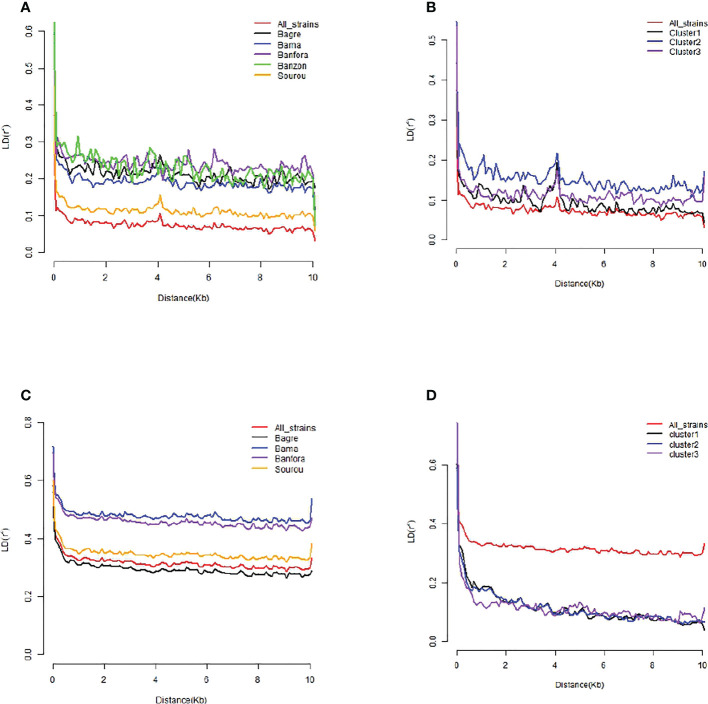
Estimated linkage disequilibrium decay (LD-decay) of *Bipolaris oryzae* and *Exserohilum rostratum* isolates. **(A)** LD of *B. oryzae* isolates per locality, **(B)** LD of *B. oryzae* isolates per cluster, **(C)** LD of *E. rostratum* isolates per locality, **(D)** LD of *E. rostratum* isolates per cluster. Localities and genetic clusters with less than 8 isolates were not presented individually (ie Banzon for *E. rostratum*, cluster 4 for *B. oryzae* and *E. rostratum*).

**Figure 7:**
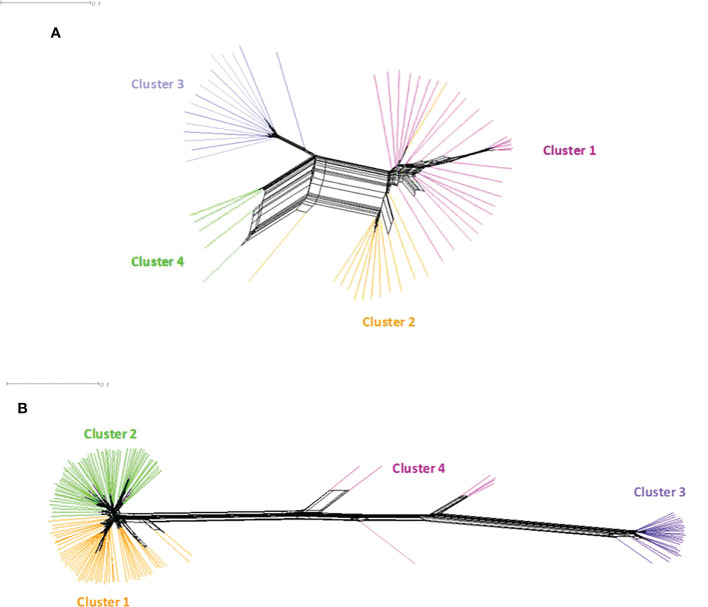
NeighborNet phylogenetic network of *Bipolaris oryzae* and *Exserohilum rostratum* isolates from Burkina Faso estimated with SPLITSTREE. **(A)** *Bipolaris oryzae*, **(B)** *Exserohilum rostratum*. Each color represents a genetic group previously defined by DAPC. For *Bipolaris oryzae*, Cluster 1 is composed of 27 isolates (Bagre = 4, Bama = 3, Banfora = 5, Banzon = 8 and Sourou =7), Cluster 2 is composed of 13 isolates (Bagre = 4, Bama = 3, Banfora = 1, Banzon = 2 and Sourou =3), Cluster 3 is composed of 16 isolates (Bagre = 1, Bama = 4 and Sourou =11), Cluster 4 is composed of 5 isolates (Banfora = 5). For *Exserohilum rostratum*, Cluster 1 is composed of 58 isolates (Bagre = 38, Bama = 4, Banfora = 4, Banzon = 2 and Sourou =10), Cluster 2 is composed of 60 isolates (Bagre = 23, Bama = 4, Banfora = 7, Banzon = 2 and Sourou =24), Cluster 3 is composed of 26 isolates (Bagre = 5, Bama = 5, Banfora = 2 and Sourou =14) and Cluster 4 is composed of 7 isolates (Bagre = 3, Banzon = 1 and Sourou = 3).

In *B. oryzae* populations from Burkina Faso, Linkage Disequilibrium (LD) pattern showed a rapid decay to less than 1 kb in all populations (**Figure 6**) suggesting high rate of recombination. The PHI coefficient calculated on the populations with sufficient number of isolates, rejected the hypothesis of clonality (p = 0.0). Finally, a reticulated NeighborNet was observed (**Figure 7A**), also supporting the hypothesis of recombination in populations of *B. oryzae* from Burkina Faso. Finally, the mating types MAT1-1 and MAT1-2 were observed in balanced proportions (**Table 7**).

**Table 7:** Distribution of mating types in *B. oryzae* and *E. rostratum* populations in five rice growing areas of Burkina Faso.

	*Bipolaris oryzae* population						*Exserohilum rostratum* population							
	N^a^	Phi test	MAT 1.1	MAT 1.2	No amplicon	p^b^	N^a^	Phi test	MAT 1.1	MAT 1.2	MAT1.1+MAT1.2^c^	No amplicon	p^b^
Localities													
Bagré	9	NA	4	5	0	NA	69	0.0	6	55	6	2	3.5 x10^-10^
Bama	10	0.0	7	3	0	0.206	13	0.0	0	12	1	0	5.3 x10^-4^
Banfora	5	NA	1	3	1	NA	13	NA	0	11	1	1	NA
Banzon	9	0.0	4	5	0	NA	5	NA	0	5	0	0	NA
Sourou	21	0.0	13	8	0	0.275	51	0.0	4	36	2	9	4.2 x10^-7^
Genetic clusters													
Cluster 1	22	0.0	11	11	0	1	58	0.0	4	46	7	1	2.8 x10^-9^
Cluster 2	13	0.0	7	5	1	0.564	60	0.0	3	56	1	0	5.2 x10^-12^
Cluster 3	16	0.0	11	5	0	0.134	26		2	13	1	10	4.5 x10^-3^
Cluster 4	3	NA	2	1	0	NA	7	NA	1	4	1	1	NA

a: Number of isolates per locality (from the same rice field) or per genetic cluster whose mating type has been characterized; b: Probability of chi-square value. For p < 0.05, the ratio of mating types deviates significantly from the null hypothesis of a 1:1 ratio. c: number of isolates amplifying of both mating types. To discard the possibility of mixed isolates, a second monoconidial isolation of the isolates amplifying both mating types was performed. Results presented here are the amplification of the subclones. NA, Not calculated due to sample size.

In *E. rostratum*, LD showed a rapid decay to less than 1 kb in all populations (**Figure 6**) and PHI coefficient rejected the hypothesis of clonality (p = 0.0), supporting the hypothesis of recombination. Low reticulated NeighborNet was observed (**Figure 7B**). The dominance (79%) of only one mating type (MAT1-2) was observed and the null hypothesis of a 1:1 ratio was rejected by statistical analysis. Ten strains repeatedly amplified markers of the two mating types (**Table 7**). Potential mixture of strains was discarded by repeating monoconidial isolation and potential contamination was discarded by repeated independent DNA extractions.

To test the sexual fertility of *B. oryzae* and *E. rostratum* isolates, crosses between isolates of opposite mating type from the same rice field were performed *in vitro*. Out of a total of 156 *in vitro* crosses for *B. oryzae*, two pairs of isolates (BF9259 and BF9268; BF9294 and BF9296) produced pseudothecia containing ascospores. Concerning *E. rostratum*, out of 364 crosses made, no pseudothecia were observed.

## Discussion

Brown spot of rice (BSR) is an emerging disease in Burkina Faso. Although it was already described, the incidence of the disease increased very recently to reach 80% of farmer’s fields from the western part of the country (Barro et al., 2021). Knowledge of the structure and genetic diversity of the fungal species predominantly responsible for the disease is an important information for disease management and to understand why the disease emerged. This study is the first comprehensive analysis of the genetic diversity of *B. oryzae* isolated from rice in Burkina Faso and the first study of *E. rostratum* populations. It is also the first study using genotyping-by-sequencing based analysis to characterize populations of *B. oryzae* and *E. rostratum*.

The *B. oryzae* and *E. rostratum* populations were sampled in five distinct rice-growing areas of Burkina Faso with an average distance of 365 km between sites (Bagré, Bama, Banfora, Banzon and Sourou). For each fungal species, the five geographic populations did not display a significant genetic differentiation. The absence of genetic differentiation among *B. oyzae* geographical populations was already observed in Bangladesh (Kamal and Mia, 2009), India (Archana et al., 2014a; Archana et al., 2014b), Iran (Nazari et al., 2015) and Thailand (Chaijuckam et al., 2019). In contrast to this situation, Burgos et al. (2013) established a relationship between the population structure and the geographical origin of isolates from different provinces in the Philippines. Although airborne spores of *B. oryzae* are important in the initiation of infections (Picco and Rodolfi, 2002; Chakraborty et al., 2020), their spatial spread seems relatively limited (about 5 m) (Chakraborty et al., 2020). It is therefore possible that the dissemination of *B. oryzae* isolates is mainly carried out by seeds. This hypothesis likely applies to *E. rostratum* that is frequently sampled from infected rice seeds (Cardona and Gonzáles, 2007; Ouédraogo et al., 2016; Silva et al., 2016; Imrani et al., 2019; Kaboré et al., 2022). In Burkina Faso there are significant exchanges of rice seed between producers across rice production areas. This agricultural practice likely supported the dissemination of *B. oryzae* and *E. rostratum* isolates across areas and could account for the lack of relationship between genetic groups and geographic origin of isolates.

The genetic diversity of *B. oryzae* has been studied using several molecular tools in some countries (Kamal and Mia, 2009; Castell-Miller and Samac, 2012; Archana et al., 2014a; Archana et al., 2014b; Nazari et al., 2015; Ahmadpour et al., 2018; Chaijuckam et al., 2019). Most of them have revealed that these populations had a high level of genetic and genotypic diversities. *Bipolaris oryzae* populations in the five rice-growing areas of Burkina Faso also displayed a high genotypic diversity, equal frequencies of MAT1-1 and MAT1-2 mating types and genomic evidence of recombination. Balanced frequencies of mating types are expected in populations of bipolar heterothallic species experiencing sexual reproduction because mating type is under the control of a single locus with two pseudo-alleles (idiomorphs). So, a 1:1 segregation of mating types is expected after sexual reproduction. All these genetic and biological characteristics are consistent with sexual reproduction. However, sexual reproduction of *B. oryzae* was seldom observed in the crosses we performed *in vitro* and pseudothecia (sexual reproductive structure) have never been observed in the field. Timing sexual reproduction phenomena remains problematic and it is difficult to know if they are still ongoing or if they occurred in the past (Castell-Miller and Samac, 2012). The hypothesis of sexual reproduction of *B. oryzae* isolates from Burkina Faso will have to be verified by repeating *in vitro* reproduction tests and actively searching for pseudothecia in rice fields.

In contrast to *B. oryzae* populations, *E. rostratum* populations showed a dominant distribution of a single mating type, MAT1-2 (79%). These results are similar to those of Kusai et al. (2016), observed on *E. rostratum* isolates from rice in Malaysia. The linkage disequilibrium and homoplasy tests did not reject the hypothesis of recombination within the *E. rostratum* populations of Burkina Faso. However, sexual reproduction of *E. rostratum* was not observed in the crosses performed *in vitro* and pseudothecia have never been observed in the field. In addition, the strong bias toward one mating type (79%) suggested that actual *E. rostratum* populations are predominantly asexual. In addition, we identified 10 isolates of *E. rostratum*, i.e. 6% of our sample that carried both the MAT1-1 and MAT1-2 idiomorphs. Such isolates with two mating types were also identified within *E. turcicum* populations from maize in China (Fan et al., 2007). Sequencing the genome of the isolates with both mating types should be used to confirm that they carry both idiomorphs (mero-diploid at these loci).

This study provides new knowledge useful to adapt disease management. First, during this study we have shown that two species are causing BSR in Burkina Faso and that *E. rostratum* is more frequently encountered than *B. oryzae.* This information is important to consider for breeding for resistance: screening of new rice varieties should be done with strains of both species. Second, the fact that there is no obvious population structure at the country suggests that there is gene flow on a broad geographic scale. We hypothesize that this is due to transportation of infected seeds. If true, a prophylactic method based on healthy seeds could have an impact. Third, the absence of geographic structure also informs on the strategy of breeding for and deployment of resistant varieties. Since the populations of both pathogenic species are not differentiated at the country scale, a resistant variety effective in a region is expected to be effective in another and selection for resistance in different regions is not mandatory. Conversely, the existence of genetic clusters in the two *B. oryzae* and *E. rostratum* probably require to screen for resistance with representative strains of these genetic groups. Finally, knowing that recombination is frequent must draw the attention on the adaptive potential of both species since favorable alleles controlling, for example, virulence to a particular resistance gene or resistance to fungicides, will easily be transmitted.

## Data availability statement

The data presented in the study are deposited in the European Variation Archive (EVA) repository, accession number PRJEB56783.

## Author contributions

KK: Collection of samples, Data production, Data analysis, Writing and proofreading AK: Collection of samples, Writing and proofreading. HA: Data production, Writing and proofreading. JM: Data production, Writing and proofreading. SG: Data production, Writing and proofreading. LB: Data production, Writing and proofreading. LC: Data production, Writing and proofreading. SR: Data analysis, Writing and proofreading. FC: Data analysis, Writing and proofreading. MB: Collection of samples, Writing and proofreading. CT: Collection of samples, Writing and proofreading. M-HL: Writing and proofreading. DT: Data analysis, Writing and proofreading. All authors contributed to the article and approved the submitted version.

## Acknowledgements

We thank the RICE CRP and the Islamic Development Bank for their financial support. This work was facilitated by the “International joint Laboratory LMI PathoBios: Observatory of plant pathogens in West Africa: biodiversity and biosafety” (www.pathobios.com; twitter.com/PathoBios). We are very grateful to the fieldworkers in Burkina Faso notably Kader Guigma, Yacouba Kone, Compaoré Abraham Terry Cedric and Hébié Bakary. We thank the technical support structures (Bagrépole and Autorité de Mise en Valeur de la Vallée du Sourou) and the rice farmers from the study areas for their kind collaboration during sampling rice leaves and seeds. We also thank Pierre Mournet and Julien Frouin for their help in the development of the GBS technique at the UMR AGAP/CIRAD genotyping platform. Part of the experiments were supported by Agropolis Fondation (Labex Agro:ANR 10 LABX 0001 01) under RiPaBIOME project (ID 1702 011).

## Conflict of interest

The authors declare that the research was conducted in the absence of any commercial or financial relationships that could be construed as a potential conflict of interest.

## Publisher’s note

All claims expressed in this article are solely those of the authors and do not necessarily represent those of their affiliated organizations, or those of the publisher, the editors and the reviewers. Any product that may be evaluated in this article, or claim that may be made by its manufacturer, is not guaranteed or endorsed by the publisher.
