# Supplementary material for: Genetic diversity and structure of Bipolaris oryzae and Exserohilum rostratum populations causing brown spot of rice in Burkina Faso based on genotyping-by-sequencing
Source: Front Plant Sci. 2022 Nov 25;13:1022348. doi: 10.3389/fpls.2022.1022348 (PMC9732276; doi:10.3389/fpls.2022.1022348)

**Supplementary Figure 4.** Relationship between geographic distance and pairwise F_ST_ of *Bipolaris oryzae* populations from five rice-growing zones in Burkina Faso


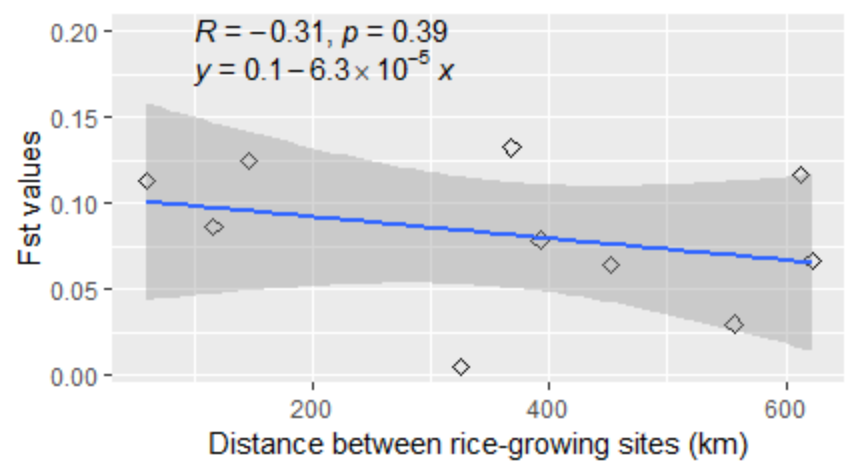

Supplement: Supplementary file 1 [file DataSheet_1.zip › Supplementary/Supplementary Figure 4.docx]
